# Multi-storm analysis reveals distinct zooplankton communities following freshening of the Gulf of Mexico shelf by Hurricane Harvey

**DOI:** 10.1038/s41598-022-12573-y

**Published:** 2022-05-24

**Authors:** Z. M. Topor, M. A. Genung, K. L. Robinson

**Affiliations:** Department of Biology, University of Louisiana at Lafayette, Lafayette, 70503 USA

**Keywords:** Biooceanography, Climate-change ecology, Community ecology, Ocean sciences, Marine biology

## Abstract

Tropical cyclones can highly modify coastal ecosystems through interactions between their unique set of meteorological traits and an ecosystem’s antecedent conditions. As such, resultant changes to biological community structure are likely storm-specific, yet our understanding of cyclone effects on marine communities is limited compared to communities in terrestrial and freshwater habitats. Using northwestern Gulf of Mexico (NWGOM) mesozooplankton data, we tested: (1) for differences between storm and non-storm community structure and dispersion; (2) if post-storm communities varied between one another; (3) if salinity drove differences; and (4) if physical drivers of abundance and evenness varied between storm and non-storm communities. Mesozooplankton community structure following Hurricanes Harvey, Ike, Rita, and during five non-storm years were analyzed. Post-Ike, post-Rita, and non-storm communities were similar while post-Harvey communities were distinct from non-storm years. A structural equation model revealed stratification and abundance drove community evenness. Post-Harvey mesozooplankton were more abundant in low salinity waters; a pattern muted during non-storm years. NWGOM mesozooplankton community structure was generally resilient to hurricane effects, except when large changes in salinity occurred. Our findings suggest resource availability for planktivorous predators and energy transfer within coastal food webs is altered following cyclones with high precipitation rates.

## Introduction

Tropical cyclones are intense meteorological events that can catastrophically affect coastal ecosystems where they make landfall. Compounding drivers such as strong winds, heavy rainfall, and ocean mixing can lead to complex ecological changes for planktonic communities following the storm, with effects that vary over space and time (days to months^1,2^). Variation in ecological response to a tropical cyclone is typically attributed to either an endogenous driver like changes in prey composition^3^, or an exogenous one, such as changes in salinity from a freshwater pulse^4^. Yet, it has recently been shown that both the initial environmental context (e.g., water column stratification^5^), and the unique characteristics of each storm (e.g., max wind speed, total precipitation^6^) are critical. This is particularly true for interpreting post-cyclone changes in plankton community structure, abundance, and ecosystem function^5–7^. For example, when pre-storm waters of the Neuse River Estuary were stratified, estuarine phytoplankton bloomed following tropical cyclone Helene. In contrast, no change in overall chlorophyll-α levels were recorded after Hurricane Alex—which was attributed to the estuary being well-mixed prior to the storm^5^. Careful consideration of an ecosystem’s antecedent conditions (sensu^6^) is therefore necessary when analyzing the ecological impacts of tropical cyclones, especially within the same ecosystem.

An average of 14 tropical systems develop each year in the Atlantic Basin^8^. Highly active hurricane seasons (e.g., May–Nov. 2020) may have 20 or more named storms. The northwestern Gulf of Mexico (NWGOM), specifically the Louisiana–Texas shelf (LATEX) shelf, receives many of these storms upon landfall. Situated south of Houston (Texas, USA), Galveston Bay is the seventh largest estuary in the United States and a major shipping port. This area has been subject to many tropical cyclones in the last 2 decades, with disturbance to coastal ecosystems varying by degree and type. In 2005, Hurricane Rita hit the Louisiana–Texas border on September 24th as a Category 3 storm; but Galveston Bay, located 160 km southwest, encountered only a mild drop in water levels and which returned to pre-storm values within 48 h^9^. Rita’s effect on the northwestern GOM zooplankton community is mostly unknown. In the central Gulf of Mexico, total chlorophyll-a concentrations were elevated following the passage of Rita due to shoaling of the nutricline^10^. Similar increases in primary production closer to shore would support additional zooplankton production. Tropical Storm Edouard (Aug. 2), Hurricane Gustav (Sept. 1), and Hurricane Ike (Sept. 13) all made landfall on the LATEX coast within the span of 2 months in 2008^11^. Of these, Hurricane Ike had the greatest impact. While rated as only a Category 2 storm, Hurricane Ike’s landfall was preceded by a “forerunner wave” that inundated the coast under 5 m of water for 3 days and washed-out protective dunes and barriers^12^. For the 2 days following Ike, cooler sea surface temperatures (SST) prevailed, and chlorophyll-α concentrations increased near the coast^13^. Like Rita, the effects of Hurricane Ike on NWGOM shelf zooplankton communities are poorly understood. In estuarine Galveston Bay (TX), Liu et al.^2^ found acute and significant shifts in zooplankton communities in response to Ike-related flooding. These changes included distinct communities at the upper and lower sections of the bay. Much like 2008, 2017 was an active year for cyclones in the NWGOM, with five named storms making landfall on the Gulf coast^14^. Notable for Galveston Bay was Hurricane Harvey (hereafter Harvey). Harvey made landfall as a Category 4 storm near the city of Houston on August 27th garnering national attention for its intense rainfall (92.7 billion m^3^ of in 5 days), earning the distinction as the wettest tropical cyclone ever recorded^15^. Subsequent severe flooding and loss of life ranks Harvey as the costliest storm in U.S. history with 80 fatalities and $160 billion in damages^16^.

Harvey floodwaters inundated Galveston Bay and the adjacent northwestern Gulf of Mexico reducing salinity in the bay to zero^1^. This freshening subsequently altered phytoplankton community composition^17,18^ and homogenized zooplankton communities in both space and time in the bay^2^. A large freshwater plume extended to the coastal margin of the NWGOM^19^, decreasing salinity, temperature, and phytoplankton biomass on the shelf^20^. The coastal margin showed prolonged impacts of Harvey. Water velocities at the mouth of Galveston Bay were elevated until 6 September 2017, and satellite imagery captured high levels of chromatic dissolved organic matter (CDOM) and dissolved organic carbon (DOC) exiting the bay onto the shelf. CDOM and DOC values did not return to pre-storm levels until 30 September 2017, a full month after Harvey made landfall^1,19^.

Changes in Galveston Bay mesozooplankton community structure were found up to 2 months after Harvey’s landfall^2^. However, it is unknown if shelf mesozooplankton communities were altered as well, as residence times vary considerably between environments (13 days in Galveston Bay versus < 1 day on the shelf^15,21^). Kurtay et al.^20^ reported decreases in overall phytoplankton biomass relative to historic levels and numerical increases of pico- and nanoplankton on the northwestern GOM shelf 2 months following Harvey. In the same area, Topor et al.^22^ reported higher abundances 1 month after the storm in September as well as subsequent shifts in mesozooplankton abundance and diversity relative to the 6 months following the storm. A caveat to these findings is that NWGOM zooplankton abundance is highly variable year-to-year^23,24^, and these results must be placed within historic context to determine if Harvey was indeed anomalous^20,22^.

Event attribution studies and physics support with high confidence the hypothesis that anthropogenic climate change has increased heavy precipitation associated with tropical cyclones and that this driver contributed to Harvey’s extreme rainfall^25^. As the Atlantic Ocean and the Gulf of Mexico continue to warm in response to global climate change, the increased heat content of the oceans will favor the formation of heavy rainfall hurricanes like Harvey^26,27^. Broader-scale meta-analyses have yielded projections that intense hurricanes (defined by wind speed) will become more frequent in the Atlantic Ocean basin with global climate change^25,26^. Slower, wetter storms will likely result in greater freshwater discharge to coastal oceans from flooding, altering salinity regimes and sediment loads to the waters of the shelf, while increased wind speeds will amplify subsurface mixing of the water column, disrupting the physical and chemical environment through changes in temperature and nutrients. All of these biophysical mechanisms are known to independently affect coastal zooplankton communities^4,28,29^, but it has yet to be shown how zooplankton react to the compounding influence of these intensified tropical cyclones.

**Figure 1:**
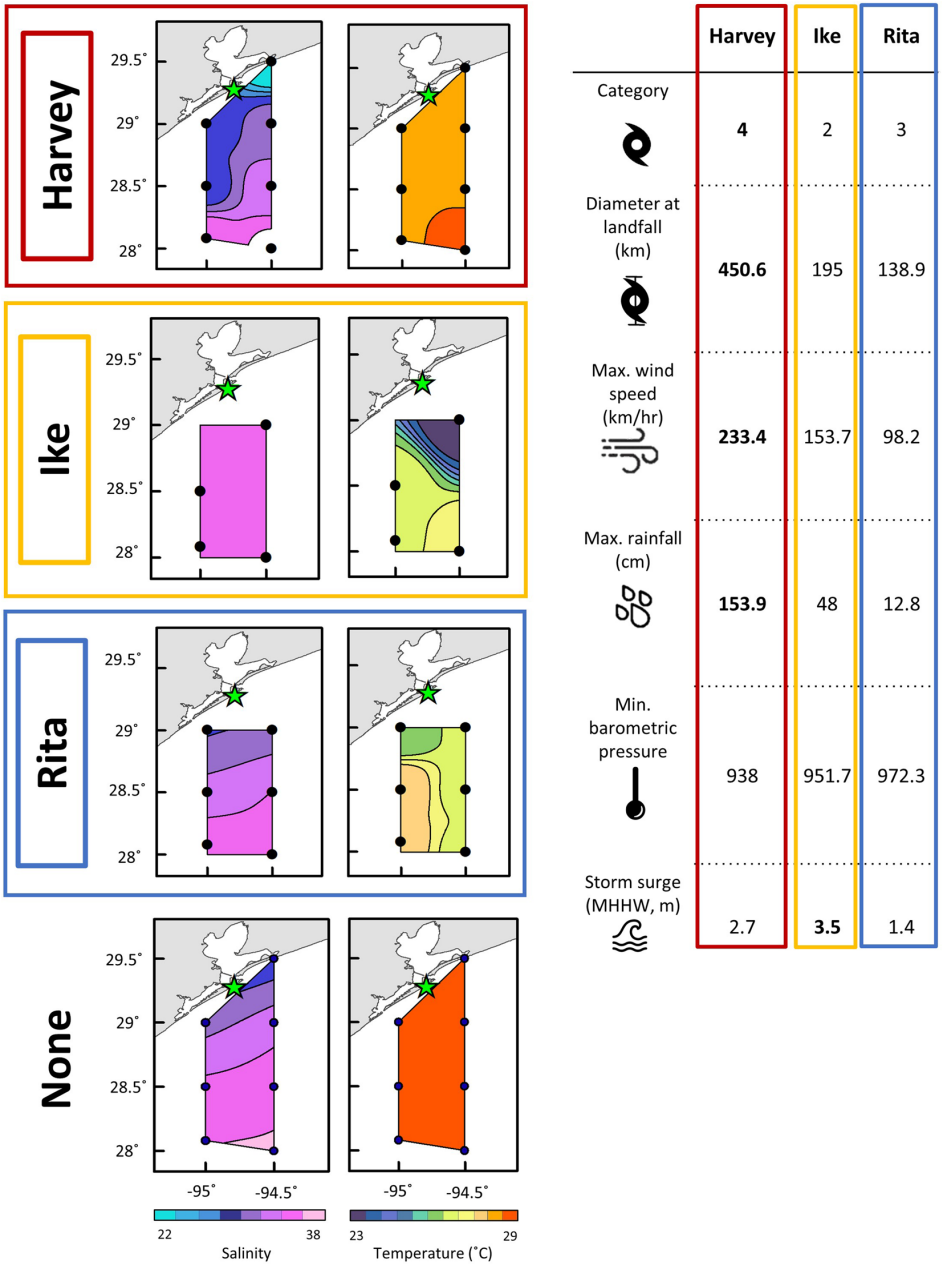
Spatial patterns of salinity (left, psu) and temperature (right, °C) recorded following Hurricanes Harvey (red), Ike (yellow), and Rita (blue) in the NWGOM off the coast of Galveston Bay, Texas (green star) compared to ‘non-storm’ conditions (bottom). Unique characteristics of each cyclone are displayed adjacent with maximum values in bold.

Using a single study area and considering the characteristics of each storm (each storm varies along a wind speed and precipitation gradient, Fig. 1), we tested four hypotheses about how mesozooplankton community structure and abundance respond to tropical cyclones in the northwestern GOM. Specifically, we predicted that: (1) post-storm mesozooplankton community structure would differ from non-storm communities and their dispersion would be greater; (2) post-storm communities would be dissimilar; (3) salinity would be the major driver of structural differences between storm and non-storm communities; and, (4) the strength of relationships between environmental drivers and mesozooplankton abundance would differ between storm and non-storm years.

## Methods

We used mesozooplankton samples collected across 12 years (2005—2017) between September–November in the northwestern Gulf of Mexico (Suppl. Table 1). Samples prior to 2017 were collected as part of the Southeast Area Monitoring and Assessment Program (SEAMAP) Fall Plankton Survey. Seven established stations follow an inshore to offshore transect off the coast of Galveston Bay, Texas (USA) (Fig. 1). We included samples from years that experienced tropical cyclones (Hurricane Rita, 2005; Hurricane Ike, 2008; Hurricane Harvey, 2017) as well as 5 years that had no major storm events (2006, 2009, 2010, 2012, 2016). Storm years were represented by 29 samples (6 for Rita, 9 for Ike, and 14 for Harvey) while non-storm years had 36 samples. Post-storm samples were collected 9–53 days following the storm’s landfall (Suppl. Table 1). Each hurricane made landfall along the Texas and Louisiana coastline and were classified as ‘severe weather events’ for the Houston area by Texas A&M’s State Climatologist^30^. Zooplankton sampled after Hurricane Harvey in 2017 were collected at the established SEAMAP stations during September (21–24 Sept), and October–November (30 Oct–3 Nov) following Topor et al.^22^.

### Mesozooplankton

Physical SEAMAP mesozooplankton samples (pre-2017) were obtained from NOAA National Marine Fisheries Service Plankton Archive. During Oct. 2017, a 1-m^2^, 333-µm mesh Multiple Opening/Closing Net Environmental Sampling System (MOCNESS) was used at stations with water depths > 5 m. A single MOCNESS net was towed to 2 m above sea floor in water depths < 100 m or to 100 m at depths ≥ 100 m to the ocean’s surface. As the initial net of the MOCNESS integrates the water column fished, these methods allowed us to compare data between gear types. All samples collected under the SEAMAP program (including Sept. 2017) were collected with a 0.5 m diameter, 335-µm mesh bongo net towed obliquely from 2 m above sea floor to the ocean’s surface and preserved in 5% buffered formalin-seawater solution^31^. For both the MOCNESS and bongo tows, the bottom third of all nets were washed from the outside into a 5-gallon bucket. The sample was then filtered through a 200-µm mesh sieve to concentrated it into a 1-L bottle. All samples were preserved and stored in 5% buffered formalin and seawater solution.

Mesozooplankton were identified and enumerated following Topor et al.^22^. Physical samples were homogenized in a graduated beaker using an aquarium bubbler and multiple aliquots (2 mL or 5 mL) were taken using a Henson–Stempel pipette. The total number of aliquots varied based on the sample volume such that the total counts reached ≥ 500 individuals/sample. The homogenized sample was then imaged with a Zooscan benchtop plankton imaging system (Hydroptic v3 ZSCA04^32^). Individual organism vignettes (> 200 µm) created by the ZooProcess software were manually classified to one of 27 taxonomic orders by trained experts on the EcoTaxa platform (https://ecotaxa.obs-vlfr.fr/). Taxa-specific density was standardized in each sample using Eq. (1):

1 $$D = \frac{N}{{P_{T} \times V}}$$

where *D* is the density of organisms (individuals/m^3^), *N* is the number of organisms counted from all splits, *P*_*T*_ is the proportion of the total sample used, and *V* is the total volume of water filtered (m^3^).

### Physical data

Temperature (°C), salinity, and fluorescence (µg/L) were measured between 0–100 m at each station using a SEABIRD (9 plus; SBE 11) CTD**.** To isolate the influence of freshwater lenses and surface mixing for statistical analysis, temperature and salinity were averaged over the top 5 m of the water column. To consider the productivity of the water column within the model, a value of maximum fluorescence was calculated by averaging the top five values recorded over the depth of the water column sampled by the bongo tow. We calculated the average Brunt Vaisala frequency as an index of water column stratification using the *gsw_Nsquared* function in the *gsw* package^33^. Distance to the mouth of Galveston Bay (29.36°N, − 94.74°W) was calculated using *geodDist* function in the *oce* package to create a distance from shore metric. All packages and analysis were conducted in R version 4.1.0.

### Statistical analysis

Compositional differences in mesozooplankton communities between storm and non-storm years (n = 65) were statistically differentiated using a permutational multivariate analysis of variance (PERMANOVA; 999 permutations). Community dissimilarity matrices were calculated using the Bray–Curtis index on log-transformed zooplankton abundances via *decostand* following Anderson^34^ to account for large variation in inter-cruise abundance levels. A permutational multivariate dispersion (PERMDISP) test was used to confirm equal dispersion between storm and non-storm years^34^. We followed our comparison of storm and non-storm years with a *pairwise.PERMANOVA* post-hoc test to distinguish statistically different storms. To evaluate the identity of taxa driving these differences, a similarity percentage (SIMPER) analysis was performed on the groupings found to be statistically distinguishable.

Environmental drivers of storm and non-storm years were evaluated using a Principal Components Analysis (PCA). Temperature, salinity, fluorescence, stratification, and distance from shore (km) were included in the PCA. Due to sensor malfunctions in the field, we imputed missing fluorescence values using the *imputePCA* function following the protocol of Borcard, Gillet and Legendre^35^. However, four observations (Cruise 0284, B218; Cruise 0804, B217; Cruise 0705; B219, B220) had no associated environmental data from which fluorescence could be imputed. As a result, these observations were removed from subsequent analyses. We determined principal component axes using the coefficients of the linear combination of variables stored in the *prcomp* object. We compared PC values to the predicted sum of squares, assuming all parameter contributions were equal (Sum of Squares = 0.447), deeming absolute parameter values above this cut-off meaningful.

We explored how physical parameters impacted multivariate zooplankton community structure using a second PERMANOVA analysis, with the physical parameters as continuous predictors. Following the results of our initial pairwise-PERMANOVA, we included a binary contrast of ‘Harvey’ versus ‘No Storm’ in our model. Removing Ike and Rita years, and four zooplankton samples that lacked environmental data, resulted in 51 samples being included in the PERMANOVA (permutations = 999). To circumvent collinearity between environmental predictors and independence of observations between years, we used the coefficients from the first three principal components as predictors in our second PERMANOVA. As above, our response was a Bray–Curtis dissimilarity matrix calculated from log transformed zooplankton abundances via *decostand*. All permutations of parameters and interactions between parameters were compared using the lowest Akaike information criterion for small sample sizes (AICc). AICc values were calculated using the *AICc_PERMANOVA* function^36^.

Finally, we conducted a piecewise structural equation model (PSEM) to visualize direct and indirect relationships between environmental parameters, mesozooplankton abundance, and mesozooplankton evenness. Stratification, fluorescence, and abundance were all log transformed to account for non-normality of the data. Salinity values were heavily left-skewed and were transformed by subtracting values from the maximum and then taking the natural log. This transformation requires that any parameter estimates for the effects of salinity be interpreted as if they had the opposite sign; we do this throughout the results. Because we were interested in the mechanistic drivers of storm impacts on zooplankton rather than unmeasured variables that covary with space and time (e.g., seasonal currents), we used a residuals approach to the PSEM. As such, for all variables in the PSEM, a linear model was run with distance from shore (Galveston Bay) and day of year as predictors. We then used the residuals of these models within our PSEM. Linear mixed effect models were used to build the PSEM, with ‘year’ held as a random effect to account for high interannual variability in NWGOM zooplankton community structure^24^. The model was refined by repeatedly removing the least statistically significant path unless: (1) the path was significant in the *multigroup* analysis (which would indicate that the path varied after Harvey and during non-storm years; see next sentence) or (2) the removal of the path increased the AICc for the PSEM (which would indicate that the path has enough predictive power to be worth the complexity it adds to the model). Contrasts within the model during the year of Hurricane Harvey and non-storm years were compared using the *multigroup* function in the piecewise-SEM package. This function tests whether the predictor-response relationship for any path differs between the Harvey year and other years. We used an alpha-value (*α*) of 0.05 in the multigroup analysis.

## Results

Our PERMDISP analysis showed no difference in multivariate dispersion among the storm groupings (PERMDISP; p = 0.64). Mesozooplankton community structure differed between storm (i.e., Harvey, Rita, and Ike) and non-storm years when all data were considered (PERMANOVA, p = 0.001). Pairwise comparisons among storms indicated that Hurricane Harvey drove this pattern, and other storms (i.e., Ike, Rita) were not distinguishable from non-storm years, nor from one another (Table 1). For this reason, subsequent analyses focused on ‘Harvey’ versus ‘non-storm contrasts, with data from ‘Ike’, and ‘Rita’ years omitted.

**Table 1:** Pairwise Permutational Multivariate Analysis of Variance (PERMANOVA) of natural log transformed mesozooplankton abundances. Grouping variables are based on presence of a major hurricane before sampling that year (Hurricane Rita, 2005; Hurricane Ike, 2008; Hurricane Harvey, 2017; non-storm years 2006, 2009, 2010, 2012, 2016). Bolded text indicates significance at α = 0.05.

	DF	Sums of squares	Mean sum of squares	F.Model	R^2^	p-value
Harvey vs. Ike	1	0.011	0.011	0.856	0.039	0.457
Harvey vs. Rita	1	0.014	0.014	0.883	0.047	0.395
**Harvey vs. None**	**1**	**0.026**	**0.026**	**2.465**	**0.049**	**0.041**
Ike vs. Rita	1	0.006	0.006	0.435	0.032	0.655
Ike vs. None	1	0.019	0.019	1.972	0.044	0.082
Rita vs. None	1	0.010	0.010	0.930	0.023	0.434

**Figure 2:**
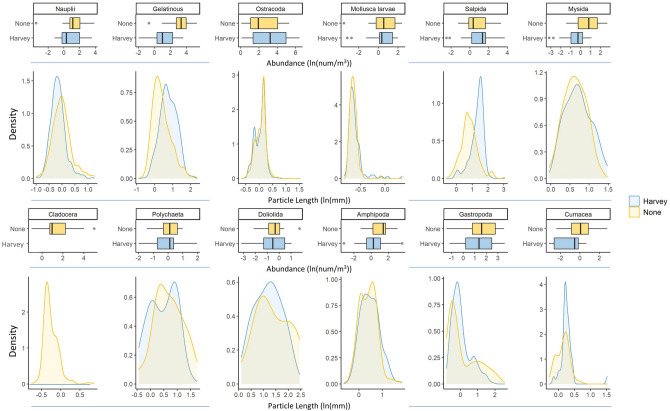
Standardized abundance (num. indv./m^3^) and maximum particle length distributions (mm) displayed for the 12 taxa determined to explain 57% of the dissimilarity between Harvey (blue) and non-storm (yellow) years (SIMPER analysis). Note that axis scales are not uniform to allow intra-taxa comparisons over Harvey and non-storm years.

Between Harvey and non-storm years, our SIMPER analysis determined that 12 taxa explained over 50% of the community structure differences found by our initial PERMANOVA. In order of decreasing percentage of community dissimilarity explained, Crustacea nauplii, Cladocera, Ostracoda, mollusk larvae, Mysida, Salpida, polychaetes, Doliolida, gelatinous zooplankton, Amphipoda, gastropods, and Cumacea all contributed to differentiating zooplankton community structure between Harvey and non-storm years. However, only Cladocera, gelatinous zooplankton, and Doliolida were found to be significant based on 999 permutations. This should be interpreted with caution as SIMPER analysis can be highly variable, and as such all taxa explaining the first 57% of variance are included in our figure (Fig. 2). Of the twelve taxa selected by the SIMPER analysis, all but Ostracoda decreased in overall abundance after Harvey. Largest relative decreases in abundance were found in gelatinous, mysids, cumaceans, and amphipods. Cladocera exhibit the greatest decreases as they were completely absent in post-Harvey samples. Body sizes of hard-bodied taxa remained relatively consistent between Harvey and non-storm years (e.g., mysids, amphipods). However, gelatinous and salps exhibited marked shifts in their distribution towards increased size following Harvey.

**Figure 3:**
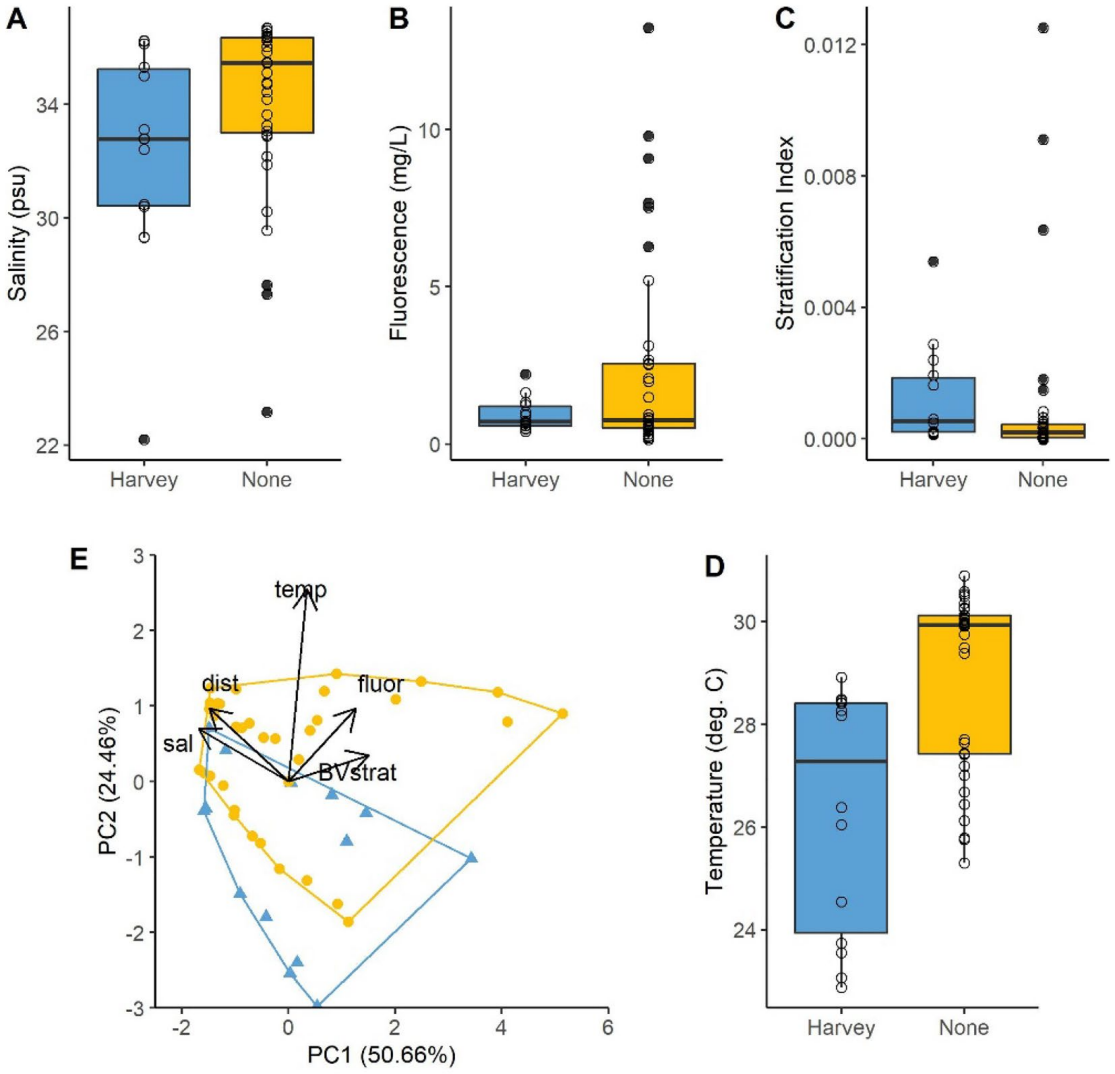
Distribution of (**A**) salinity, (**B**) fluorescence, (**C**) temperature, and (**D**) stratification index between Hurricane Harvey and non-storm years. (**E**) PCA of environmental observations (n = 62). *Sal* salinity (psu), *fluor* fluorescence (µg/mL), *temp* temperature (°C), *BVstrat* stratification index, *dist* distance from shore (km).

Three principal components synthesizing environmental conditions during Harvey and non-storm years explained 91% of the total variance (Fig. 3; PC1: 50.66%, PC2: 24.46%, PC3: 15.92%). Salinity, fluorescence, and temperature were all lower during Harvey relative to non-storm years (Fig. 3A,B,D), while stratification was slightly higher, yet less variable during Harvey (Fig. 3C). PC1 was characterized by a strong negative relationship with salinity, a slight positive relationship with stratification, and a negative relationship with distance from shore (Table 2). PC2 was strongly positively related to temperature. PC3 had a strong positive relationship with water column stratification and strong negative relationship with fluorescence (Table 2).

**Table 2:** Coefficients of the first three axis from the principal component analysis. Cumulative proportion of variance explained with the first three axis equals 90%. Bolded values represent absolute values exceeding the predicted sum of squares, assuming all equal parameter contribution (0.447).

	PC1 (50.66%)	PC2 (24.46%)	PC3 (15.92%)
Salinity	− **0.563**	0.234	− 0.258
Temperature	0.116	**0.850**	0.089
Stratification	**0.499**	0.117	**0.605**
Fluorescence	0.420	0.322	− **0.646**
Dist. from shore	− **0.494**	0.324	0.377

The most parsimonious environmental PERMANOVA included the additive combination of PC1 (positive stratification, negative salinity, and distance from shore) and PC3 (positive stratification, negative fluorescence) with an interaction between PC2 (positive temperature) and the Harvey contrast (AICc = − 164.84; Table 3). Within the accepted range of Δ2 AICc, all models included PC1, PC2, and the Harvey contrast; models varied most by the inclusion and identity of term interaction (Suppl. Table 2). PC3 was included in two of the top five models but was never included in an interaction.

**Table 3:** Results of the top PERMANOVA (n = 48) chosen by AICc. PC1, PC2, and PC3 are coefficients from principal coordinates analysis and are described in Table 2. ‘Harvey’ represents a binary contrast of Harvey v non-storm years. Significant predictors of zooplankton community structure are bolded (α ≤ 0.05).

	Df	Sum of Squares	R^2^	F-statistic	p value
**PC1**	**1**	**0.155**	**0.095**	**5.620**	**0.001**
**PC2**	**1**	**0.074**	**0.045**	**2.689**	**0.012**
**Storm**	**1**	**0.111**	**0.068**	**4.036**	**0.001**
**PC3**	**1**	**0.070**	**0.043**	**2.551**	**0.016**
**PC2:Harvey**	**1**	**0.069**	**0.043**	**2.526**	**0.018**
Residual	42	1.155	0.707		
Total	47	1.634	1.000		

**Figure 4:**
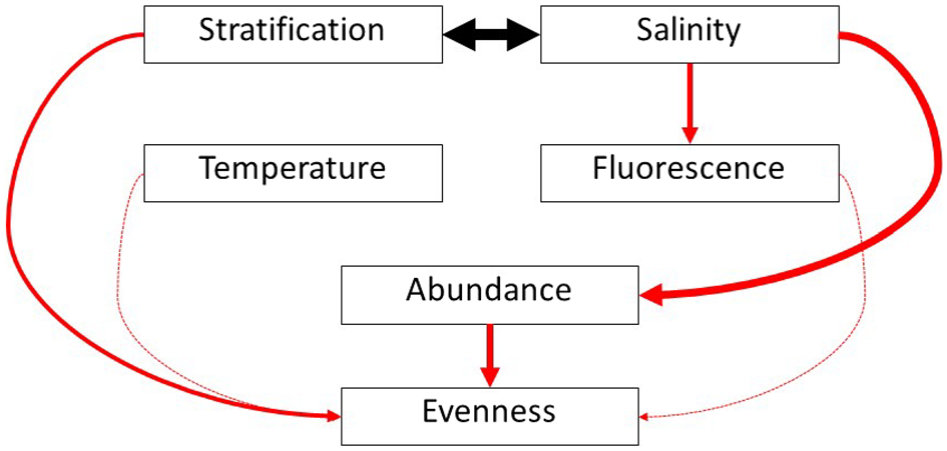
Structural Equation Model (PSEM) of zooplankton abundance and evenness in the northwestern GOM. Red arrows indicate negative relationships between parameters, while black hollow-headed arrows represent positive. Relative size of the arrow width indicates magnitude of the interaction. Dotted lines indicate cases in which there was a non-significant main effect of the predictor on the response variable, but the predictor interacted with “Harvey” to affect the response variable.

The best piecewise structural equation model (PSEM) of environmental effects on mesozooplankton abundance and evenness revealed that higher total mesozooplankton abundance and water column stratification was directly related to lower community evenness (Fig. 4). Stratification and salinity were strongly related to one another, and fluorescence decreased with increasing salinity (Fig. 4).

**Figure 5:**
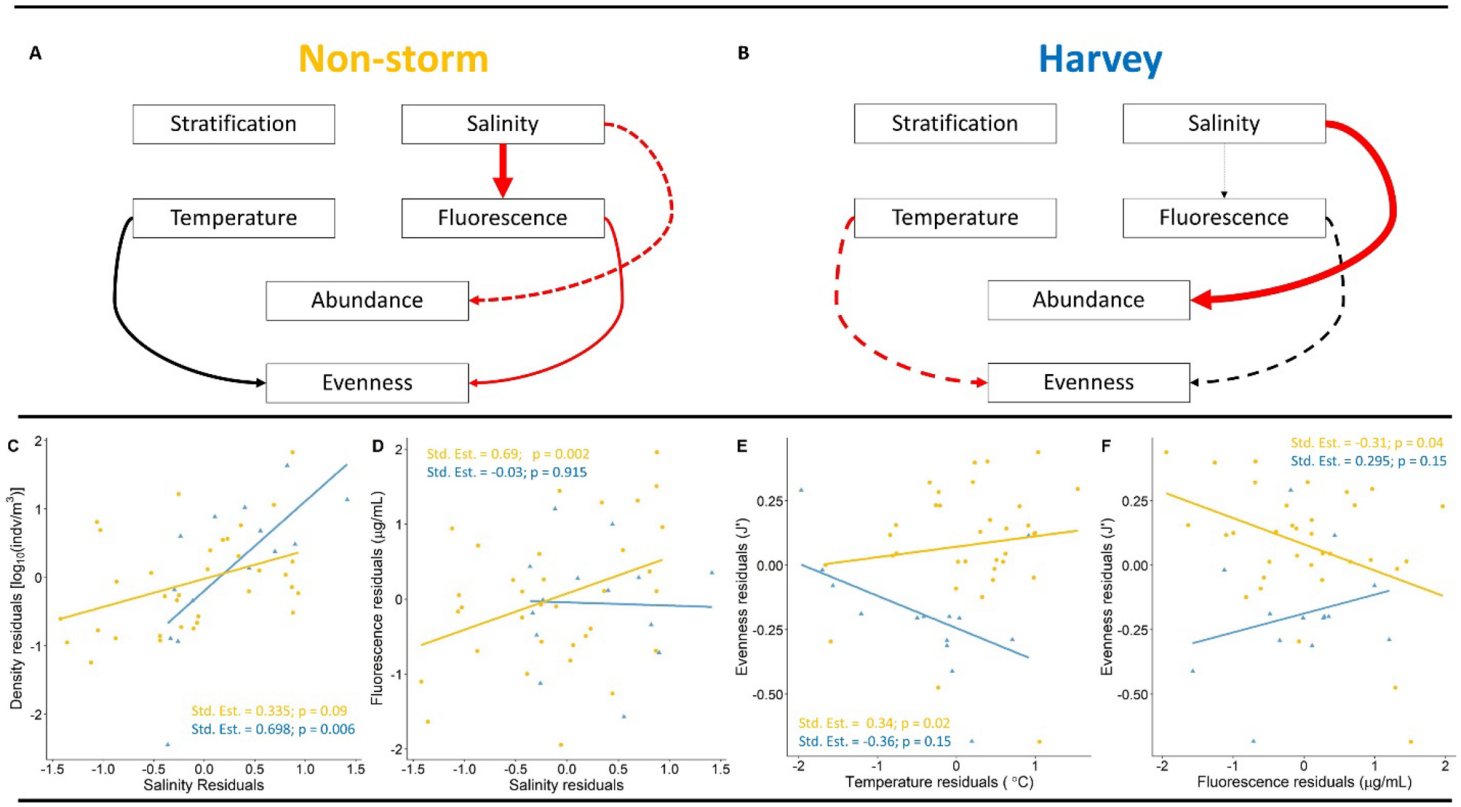
Multigroup PSEM of Harvey and non-storm models. Panels below break out changes in relationship highlighted by the multigroup analysis. Line color and point shape represent Harvey (blue triangles) and non-storm (yellow dots) samples. Red arrows indicate negative relationships between parameters, black arrows mean positive. Relative size of the arrows indicates magnitude of the interaction.

The PSEM contrasts between Harvey and non-storm mesozooplankton abundance and community evenness values revealed major changes in the directionality and standardized parameter estimates of some relationships (Fig. 5). Salinity strongly decreased fluorescence during non-storm years but had a non-significant positive effect on fluorescence following Harvey. Similarly, higher fluorescence during non-storm years led to lower evenness, but was not significantly related during Harvey. Temperature increased mesozooplankton evenness during non-storm years, but had a non-significant negative effect on evenness following Harvey. In contrast, salinity strongly decreased abundance following Harvey, but had a smaller, non-significant effect on abundance during non-storm years (Fig. 5).

## Discussion

In this study, we aimed to determine if tropical cyclones in the northwestern GOM differentially affected mesozooplankton community structure. We found that multivariate community structure varied between storm and non-storm years. However, among the three hurricanes, only the post-Harvey mesozooplankton communities were distinct from years where no storms occurred. Multivariate dispersion, a measure of variance within community structure, did not differ between storm and non-storm years. This result refutes our hypothesis that variability in zooplankton community structure would be higher during storm years opposed to non-storm years. Our prediction that post-storm mesozooplankton communities would differ from non-storm communities was supported, as was our expectation that mesozooplankton community structure varied among storms. We hypothesized that due to the major flooding and rainfall of Harvey, reduced salinity would likely be the main driver of mesozooplankton community differences relative to non-storm years. This expectation was partially met; differences in mesozooplankton community structure between Harvey and years with no storms were driven by salinity, stratification, and ‘distance-from-shore’ rather than solely salinity. This indicates that NWGOM zooplankton community structure varies holistically with biophysical conditions rather than being primarily driven by one or two dominating factors^24^. Moreover, we found that the presence of Hurricane Harvey, rather than temperature, explained the second greatest amount of variance in zooplankton community structure reflecting the importance of considering how complex disturbance mechanisms might compound and result in a unique ecological responses following a tropical cyclone. The overall PSEM showed that high salinity was directly associated with reduced fluorescence and depressed zooplankton abundance. Higher abundances were found to result in higher community dominance (i.e., lower evenness). Conversely, high salinities indirectly reduced zooplankton evenness via greater water column stratification. Lower fluorescence at higher salinities supports the spatial patterns in NWGOM phytoplankton identified by Kurtay et al.^20^ following Hurricane Harvey. Those authors observed declines in overall phytoplankton abundance and communities increasingly dominated by cells < 20 µm in size waters along a coastal to oceanic gradient.

Because Ike and Rita years were indistinguishable from either Harvey or non-storm years, we focused on the statistically supported contrast of Harvey and non-storm years to investigate differences in zooplankton community structure. Our expectation was that we would find a larger effect size between salinity, mesozooplankton abundance, and community evenness following Harvey. This hypothesis was partially supported. The PSEM “multigroup” analysis showed that relationships between salinity and primary production (i.e., fluorescence) switched signs and was reduced by an order of magnitude after Harvey. This reflects the post-Harvey shift in underlying phytoplankton community, towards smaller heterotrophic organisms reported by Kurtay et al.^20^. Finally, we see that after Harvey, higher temperatures led to lower zooplankton community evenness whereas the reverse was true for non-storm years.

We found that increased zooplankton food availability (i.e., fluorescence) was linked to a decrease in zooplankton community evenness during non-storm years but, this connection was greatly weakened and the direction changed during Harvey, likely due to the overall reduction in fluorescence^20^. The reduction of zooplankton evenness at high productivity during non-storm years may be explained by the “paradox of enrichment”, where excessive resource availability (e.g., high productivity) can result in specialization, leading to dominance of a few taxa, reducing community diversity and evenness^37^.

Notably, our model did not link fluorescence and mesozooplankton abundance in either the full or multi-group PSEM. The absence of this connection is likely due to the coarse summary of fluorescence as a metric for productivity, in which all photosynthetically active producers are grouped together. Producers are highly diverse, both taxonomically and functionally (e.g., size), with a wide range of habitat preferences. This diversity creates variation in the spatial and temporal resource environment for grazing mesozooplankton (e.g., pico- vs. nanoplankton^20^). Additionally, heterotrophic microzooplankton may constitute a majority of mesozooplankton diet, yet these organisms are not detected via chlorophyll fluoresced metrics.

The influence of temperature and salinity on zooplankton abundance and evenness between Harvey and non-storm years varied substantially. Confirming our hypothesis that salinity would be the major influence on zooplankton abundance, post-Harvey zooplankton abundances declined significantly more quickly with increased salinities compared to non-storm years. It is important to note that this pattern is found when spatial covariation is isolated, essentially removing the known influence of distance from shore gradients on salinity and abundance. We attribute the switch in directionality and significance of temperature on evenness between Harvey and non-storm years to the compressed temperature range exhibited during Harvey. This compression reduced the influence of temperature on zooplankton dominance resulting in the negative, non-significant relationship shown. These results indicate that cyclones like Hurricane Harvey can affect northwestern GOM zooplankton communities via changes in salinity, either through direct effects on zooplankton abundance or indirectly through the reduced influence of fluorescence on zooplankton evenness.

Tropical cyclones represent large coastal disturbances, and it is expected that years during which large storms pass through an area, community composition and structure of the biologic constituents will differ from years of no cyclone disturbance^2,5,17^. We found that presence of a storm significantly predicted differences in mesozooplankton community structure relative to non-storm years, supporting our original hypothesis. However, we surprisingly found that only post-Harvey zooplankton communities were statistically distinguishable from non-storm years. This lack of variation may be attributed to the unique characteristics of each storm included in our analysis. Hurricanes Ike and Rita both altered the coastal environment of Louisiana and Texas, but largely through different mechanisms than Harvey. Hurricane Ike, a Category 2 storm, pushed cooler, saltier ocean water into the coastal environment via a wave of storm surge greater than either Harvey or Rita. Hurricane Rita had similar wind speeds and storm surge to Harvey, but its precipitation rate was significantly less, falling between Harvey and Ike. Harvey had the highest recorded wind speeds as a Category 4 storm at landfall, and its rainfall totals far exceeded the precipitation of Ike and Rita, causing major flooding of the Houston area, drastically changed the salinity, turbidity, and temperature of Galveston Bay and adjacent coastal NWGOM^1,17,19^.

These differences in disturbance mechanism (e.g., precipitation, storm surge, mixing) and intensity may explain the variance in zooplankton communities between storm and non-storm years, but the environmental baseline remains an important consideration as antecedent conditions of a system have been shown to dictate community response after a disturbance^5^. While salinity values were significantly reduced relative to historic years following Harvey^20^ (both at the mouth of Galveston Bay and nearshore), we show that salinity values across the continental shelf do not vary considerably between Harvey and the non-storm years in our data set. Rather, we see reduced temperature and fluorescence, and increased water column stratification following Harvey. We found that salinity, fluorescence, and distance from shore (i.e., PC1), explained the most variance in community structure in our PERMANOVA, and whether or not zooplankton were collected after Harvey followed closely behind as the second strongest predictor. These conditions indicate that drivers of zooplankton community change after large cyclones like Harvey are not always obvious, and both storm characteristics and natural variability of a system must be accounted for when investigating the ecological consequences of cyclones (sensu^6^).

The ambiguity of which physical driver best describes zooplankton community structure in the northwestern GOM is unsurprising as these organisms are poikilothermic drifters that exhibit an incredible diversity of life history traits and environmental tolerances. This variety is reflected in the multitude of taxa found to influence differences in zooplankton community structure between Harvey and non-storm years from our SIMPER analysis. When we compare the abundances of taxa between Harvey and non-storm years, we see a general reduction across taxa such as amphipods, gastropods, cumaceans, mysids, polychaetes, and gelatinous zooplankton. An interesting result is the conspicuous absence of cladocerans following Hurricane Harvey. Cladocera taxa are often used as disturbance indicators in freshwater and coastal systems^38,39^ and their absence may reflect the strong environmental changes brought on by Harvey in the NWGOM as has been shown after other tropical cyclones (e.g., Hurricane Agnes^40^). Cladocera are known prey items for larval fish in this region (e.g., *Cynoscion nothus, Thunnus thynnus*^41,42^), and disturbances such as tropical cyclones may disrupt the temporal availability of this food source, leading to decreased fitness and recruitment.

While overall abundance often dictates the productivity of zooplankton communities, it is also pertinent to consider the size structure of taxa. Body size has been deemed the master functional trait in zooplankton ecology, as almost all ecological interactions and rates are intimately related to how large or small a zooplankter is^43^. Zooplankton body size may also influence the structuring of higher trophic levels through prey suitability. Larval fish predation on zooplankton is size-limited, constrained by maximum gape width or gill raker density^41^, while gelatinous filter feeders such as salps, doliolids and appendicularians are limited by their mesh size. While we see generally strong overlap in size distribution between Harvey and non-storm years, few taxa, such as doliolids, salps, and gelatinous zooplankton show a greater size after Harvey, though their abundance is decreased or equivocal to non-storm years (Fig. 2). Changes in the abundance of larger, soft bodied organisms such as gelatinous zooplankters may alter the available carbon within coastal food web and redirect secondary production through the microbial loop rather than transferring energy to higher trophic levels. Additionally, filter feeding gelatinous zooplankton such as doliolids and salps can process and filter greater volumes of water at increased body sizes^44,45^, potentially increasing their ecological success leading to positive feedback of recovering energy from the microbial loop^46^ and enhancing vertical carbon flux^47^. Kurtay et al.^20^ reported an increase in pico- and nanoplankton, prey items that falls within the filtering size of both doliolids and salps^44,45^. The increase in their available prey after Harvey may have resulted in our observed increase in gelatinous filter feeding size. Taxa specific responses to tropical cyclones are of high interest, as different organisms within the zooplankton community react differently to environmental disturbances. Understanding which disturbance mechanism relates to which specific taxa is beyond the scope of this study, but it is an important avenue to pursue if we are to better understand long-term food web consequences within coastal ecosystems.

Holistic approaches towards understanding disturbance mechanisms, such as community structure, provide a complex picture of taxa-specific responses and compounding environmental predictors. However, when we collapsed zooplankton community variability into a univariate metric (i.e., zooplankton abundance and evenness), a clearer relationship between environmental drivers and our zooplankton emerged. Mesozooplankton evenness was largely influenced by stratification and mesozooplankton abundance. Additionally, salinity indirectly impacted evenness through water column stratification. As salinity decreases, stratification increases, reducing the overall evenness of zooplankton. Zooplankton abundance was negatively related to both salinity and zooplankton evenness. This relationship is highlighted through a major shift in the influence of salinity on mesozooplankton abundance after a high precipitation storm event (i.e., Hurricane Harvey). The overwhelming influence of salinity on mesozooplankton abundance following Harvey, but not during non-storm years, emphasizes the importance of unique storm characteristics and the difference in their mechanistic impact on coastal ecosystems. Within the current climate regime, larger, wetter storms are likely to occur in the Northern Hemisphere^25^. As we show here, increased precipitation from tropical cyclones can lead to a local change in zooplankton abundance in the northwestern Gulf of Mexico. Storms may also have far-reaching impacts on coastal zooplankton communities through their impacts on freshwater discharge upstream of coastal estuaries (e.g., Atchafalaya and Mississippi River). High precipitation events can modify alongshore transport and current patterns of coastal waters off the coast of Texas^15,21^ altering the environment and distribution of coastal zooplankton taxa. Consequently, larger, wetter cyclones can modify coastal food webs not only through salinity-controlled zooplankton abundances (Fig. 4) or compositional shifts (e.g.^4^), but via physical displacement potentially causing a spatial mismatch between plankton and their predators.

We show that Harvey affected northwestern GOM zooplankton communities for up to a month following landfall, longer than has been reported for other storms^48,49^. The extended temporal span of Harvey’s impacts on zooplankton, relative to Rita and Ike, likely has to do with the duration of post-storm coastal ocean conditions in the northwestern GOM. After Hurricane Rita, elevated water levels returned to pre-storm values within 48 h^9^. Similarly, after Ike, elevated water heights caused by the intrusion of 5 m of storm surge were recorded for 3 days after landfall^12^, and sea surface temperatures and elevated chlorophyll-α remained for 2 days before returning to pre-Ike levels^13^. In contrast, water levels of Galveston Bay were elevated for up to a week after Harvey, and salinities within the Bay did not return to pre-storm levels for over 2 months (62 days^1^). It is important to note that the residence time of Galveston Bay is around 1 month^50^. After the freshening event from Harvey’s rain, the bay acted like a low salinity reservoir to the NWGOM, exchanging saltwater via tidal pumping and extending the duration of low salinity intrusion into coastal waters^1^. This scenario is supported by prolonged levels of elevated water velocity at the Galveston Bay channel recorded until 6 September 2017 and elevated sediment flux out of the Bay until 26 September 2017, almost an entire month after Harvey made landfall^1,19^.

As energetic expressways between primary production and higher consumers, changes in the abundance and evenness of zooplankton communities impact energy transfer in coastal food webs. Large increases in coastal zooplankton abundance, like we see following Harvey^22^, can represent a flood of food resources for planktivorous consumers in the area (e.g., forage fish). Zooplankton abundance is often closely linked to larval fish recruitment success, with higher zooplankton abundances representing greater availability of prey in the water column^51^, but can also represent increased top-down control by zooplankton predating on larval fish^52^. We show that increases in zooplankton abundance drove down evenness, which may restrict energy transfer within the food web through selectivity effects. Plankton communities where only a few taxa are numerically dominant can become energetically unfavorable for trophic transfer if the dominant taxa are unpalatable for consumers^53^. Changes in taxa dominance can result from mechanistic selectivity issues for predators if prey selection is determined by gape or gill raker size (e.g., larval fish^41^). Additionally, behavioral changes of the dominant taxa (e.g., distribution within water column, diel vertical migration) could lead to an ecological mismatch in capture efficiency by predators. For fish that spawn between September and October when cyclones activity is prevalent (e.g., *Cynoscion nothus, Larimus fasciatus*^54^), the abundance and composition of zooplankton in the northwestern Gulf of Mexico play a vital role in overall fitness of the organism^41^. Further connection between storm impacts on zooplankton size, distribution, and its downstream effect on larval fish is warranted to understand how cyclones can impact commercially important fish populations.

## Conclusion

Storms that share Harvey’s characteristics (e.g., slow speeds, high precipitation) are predicted continue to form and make landfall in the northwestern GOM during the current climate regime^25,26^ and it is imperative that we begin to disentangle differences in storm drivers and consider the historic context of the systems in question. We show that mesozooplankton communities collected after Hurricane Harvey were distinct from non-storm years and that zooplankton abundance and size distributions contrasted between taxa. Cladocera were conspicuously absent in Harvey samples, likely reflecting their sensitivity to environmental changes. Gelatinous zooplankton generally decreased or maintained their abundance between Harvey and non-storm years but increased in size, which may have amplified microbial loop processes and carbon export. A combination of environmental factors, rather than solely salinity, influenced the differences in zooplankton community structure between Harvey and non-storm years. Zooplankton evenness within the northwestern Gulf of Mexico was controlled largely by zooplankton abundance, as well as through direct and indirect effects of stratification and salinity respectively. Finally, we found that salinity had a strong, negative relationship with zooplankton abundance after Hurricane Harvey, a relationship not significant during non-storm years. The increase in zooplankton abundance but decreases in community evenness after Harvey has important implications for coastal food web energy availability and transfer as future cyclones are predicted to emulate Harvey’s characteristic high rainfall.

## Supplementary Information

Supplementary Tables.

## Data Availability

The datasets used and/or analyzed during the current study available from the corresponding author on reasonable request.
