# Supplementary material for: Multi-storm analysis reveals distinct zooplankton communities following freshening of the Gulf of Mexico shelf by Hurricane Harvey
Source: Sci Rep. 2022 May 24;12:8721. doi: 10.1038/s41598-022-12573-y (PMC9130273; doi:10.1038/s41598-022-12573-y)
Supplement: Supplementary file 1 — Supplementary Tables. [file 41598_2022_12573_MOESM1_ESM.docx]

Supplementary information for *Multi-storm analysis reveals distinct zooplankton communities following freshening of the Gulf of Mexico shelf by Hurricane Harvey*

Supplementary Table 1: Metadata for samples used in analysis with storm designation. Date: date sample was collected; Cruise: “SEAMAP” and “RP1” are 2017 post-Harvey cruises (Sept., Oct.-Nov. respectively), all numeric cruises are from the historic SEAMAP repository; Station: SEAMAP station situated off of Galveston Bay, TX; Year: year sampled; Storm: named hurricane that occurred before sampling

| **Date** | **Cruise** | **Station** | **Year** | **Storm** |
| --- | --- | --- | --- | --- |
| 10/30/2017 | RP1 | B221 | 2017 | Harvey |
| 10/30/2017 | RP1 | B222 | 2017 | Harvey |
| 10/31/2017 | RP1 | B223 | 2017 | Harvey |
| 11/2/2017 | RP1 | B217 | 2017 | Harvey |
| 11/2/2017 | RP1 | B218 | 2017 | Harvey |
| 11/2/2017 | RP1 | B219 | 2017 | Harvey |
| 11/3/2017 | RP1 | B220 | 2017 | Harvey |
| 9/22/2017 | SEAMAP | B219 | 2017 | Harvey |
| 9/22/2017 | SEAMAP | B220 | 2017 | Harvey |
| 9/23/2017 | SEAMAP | B217 | 2017 | Harvey |
| 9/23/2017 | SEAMAP | B218 | 2017 | Harvey |
| 9/23/2017 | SEAMAP | B223 | 2017 | Harvey |
| 9/24/2017 | SEAMAP | B221 | 2017 | Harvey |
| 9/24/2017 | SEAMAP | B222 | 2017 | Harvey |
| 10/14/2008 | 805 | B223 | 2008 | Ike |
| 10/15/2008 | 805 | B217 | 2008 | Ike |
| 10/17/2008 | 284 | B222 | 2008 | Ike |
| 11/4/2008 | 284 | B218 | 2008 | Ike |
| 11/5/2008 | 284 | B219 | 2008 | Ike |
| 9/22/2008 | 804 | B221 | 2008 | Ike |
| 9/22/2008 | 804 | B222 | 2008 | Ike |
| 9/22/2008 | 804 | B223 | 2008 | Ike |
| 9/7/2008 | 804 | B217 | 2008 | Ike |
| 10/16/2010 | 292 | B217 | 2010 | None |
| 10/16/2010 | 292 | B223 | 2010 | None |
| 10/16/2012 | 301 | B223 | 2012 | None |
| 10/17/2012 | 301 | B217 | 2012 | None |
| 10/17/2012 | 301 | B222 | 2012 | None |
| 10/18/2010 | 292 | B222 | 2010 | None |
| 10/18/2012 | 301 | B218 | 2012 | None |
| 10/18/2012 | 301 | B221 | 2012 | None |
| 10/19/2012 | 301 | B219 | 2012 | None |
| 10/22/2010 | 292 | B218 | 2010 | None |
| 10/22/2010 | 292 | B219 | 2010 | None |
| 8/29/2009 | 904 | B217 | 2009 | None |
| 8/29/2009 | 904 | B218 | 2009 | None |
| 8/29/2009 | 904 | B222 | 2009 | None |
| 8/29/2009 | 904 | B223 | 2009 | None |
| 8/9/2016 | 1606 | B221 | 2016 | None |
| 9/1/2009 | 904 | B219 | 2009 | None |
| 9/1/2009 | 904 | B221 | 2009 | None |
| 9/13/2010 | 291 | B221 | 2010 | None |
| 9/13/2010 | 291 | B222 | 2010 | None |
| 9/14/2010 | 291 | B223 | 2010 | None |
| 9/3/2006 | 604 | B221 | 2006 | None |
| 9/3/2007 | 705 | B219 | 2007 | None |
| 9/3/2007 | 705 | B220 | 2007 | None |
| 9/4/2006 | 604 | B217 | 2006 | None |
| 9/4/2006 | 604 | B218 | 2006 | None |
| 9/4/2006 | 604 | B222 | 2006 | None |
| 9/4/2006 | 604 | B223 | 2006 | None |
| 9/5/2006 | 604 | B219 | 2006 | None |
| 9/5/2006 | 604 | B220 | 2006 | None |
| 9/9/2016 | 1606 | B217 | 2016 | None |
| 9/9/2016 | 1606 | B218 | 2016 | None |
| 9/9/2016 | 1606 | B219 | 2016 | None |
| 9/9/2016 | 1606 | B220 | 2016 | None |
| 9/9/2016 | 1606 | B222 | 2016 | None |
| 9/9/2016 | 1606 | B223 | 2016 | None |
| 10/23/2005 | 505 | B219 | 2005 | Rita |
| 10/23/2005 | 505 | B221 | 2005 | Rita |
| 10/23/2005 | 505 | B222 | 2005 | Rita |
| 10/26/2005 | 505 | B218 | 2005 | Rita |
| 10/27/2005 | 505 | B217 | 2005 | Rita |
| 10/27/2005 | 505 | B223 | 2005 | Rita |

Supplementary Table 2: Top PERMANOVA models determined by AICc values with environmental predictors and Harvey v. non-Harvey contrast. AICc: Akaike Information Criteria for small sample sizes; AIC: Akaike Information Criteria; K: number of parameters; N: number of samples

|  | **AICc** | **AIC** | **k1** | **N** |
| --- | --- | --- | --- | --- |
| sp.dist ~ PC1 + PC2 * harvey + PC3 | -164.844 | -166.893 | 6 | 48 |
| sp.dist ~ PC1 + PC2 * harvey | -164.674 | -166.102 | 5 | 48 |
| sp.dist ~ PC1 + PC2 + PC3 + harvey | -164.661 | -166.09 | 5 | 48 |
| sp.dist ~ PC1 + PC2 + harvey | -164.485 | -165.415 | 4 | 48 |
| sp.dist ~ PC1 + harvey | -164.404 | -164.95 | 3 | 48 |
| sp.dis ~ PC1 * PC2 + harvey | -163.866 | -165.294 | 5 | 48 |
